# Harmful Effects of Prescribed Opioids in Children and Adults: A Systematic Review

**DOI:** 10.3390/ph18101429

**Published:** 2025-09-24

**Authors:** Luíza Siqueira Lima, Nayara de S. da Costa, Maria Eduarda A. Galiciolli, Quelen I. Garlet, João José Joaquim, Cláudia S. Oliveira, Cristiano Matos

**Affiliations:** 1 Instituto de Pesquisa Pelé Pequeno Príncipe, Curitiba 80250-060, Brazil; luizaelima04@gmail.com (L.S.L.); costasouzanayara@gmail.com (N.d.S.d.C.); dudagaliciolli@hotmail.com (M.E.A.G.); 2 Faculdades Pequeno Príncipe, Curitiba 80230-020, Brazil; 3 Programa de Pós-Graduação em Farmacologia, Centro Politécnico, Universidade Federal do Paraná, Curitiba 815300-000, Brazil; quelen.garlet@ufpr.br; 4 ESTESC-Coimbra Health School, Instituto Politécnico De Coimbra, Farmácia, 3045-043 Coimbra, Portugal; jjj@estesc.ipc.pt

**Keywords:** prescribed opioids, toxicity, harmful effects, pediatric patients, adult patients, damage

## Abstract

**Background:** Opioids are commonly used to manage both acute and chronic pain by acting on opioid receptors in the central and peripheral nervous systems. However, concerns about their increasing prescription and misuse have emerged due to adverse effects, toxicity, and the global impact of opioid-related harm. **Objectives:** This systematic review aims to evaluate the harmful (adverse and toxic) effects of prescribed opioids on the pediatric and the general population. **Methods:** Following PRISMA guidelines, a systematic search was conducted for data from January 2011 to December 2024 across selected electronic databases (PubMed^®^, SciELO^®^, Web of Science^®^, and EMBASE^®^) using a specific search strategy with Boolean operators. Cross-sectional, cohort, and case–control designs published in English, analyzing and identifying the harmful effects of prescribed opioids in children and the general population, were eligible for inclusion. Three reviewers independently assessed titles and abstracts for eligibility, followed by a full-text review. A referee reviewer resolved discrepancies. Data extraction was performed for qualifying studies. The risk of bias was assessed by the ROBINS-I tool. **Results:** A total of 3984 papers were collected, with 1697 duplicates and 2062 non-eligible papers removed; resulting in 25 papers (112.825 patients) selected for qualitative analysis. The pediatric group experienced more harmful effects across multiple body systems (nausea and vomiting, hypotension, agitation, drowsiness/lethargy, lethargy, respiratory depression, aspiration pneumonia) compared to the general population (dominant S wave, long QTc interval, right axis deviation, seizure). **Conclusions:** Despite the data heterogeneity, this study highlights the importance of evaluating the harmful effects of opioids, particularly in pediatric patients, to assess the risk–benefit balance and health risks associated with their use. The lower number of effects in the general population may be attributed to increased pharmacological tolerance and tolerability.

## 1. Introduction

Valued for their analgesic properties, opioids are widely used to manage acute and chronic pain of varying intensities [1]. These drugs alleviate pain by modulating descending inhibitory pathways within the Central Nervous System (CNS), ultimately affecting efferent nerve endings in the Peripheral Nervous System. This effect is mediated through their agonistic action on opioid receptors, which are transmembrane proteins belonging to the G-protein-coupled receptor family. Activation of these receptors inhibits cellular mechanisms via Gi/o protein signaling [2] and mediates the actions of both endogenous opioids (such as endorphins) and exogenous opioids, such as morphine (MOR) and fentanyl (FEN) [3].

The main types of opioid receptors are mu (μ—associated with pain relief and euphoria), delta (δ—pain relief and mood regulation), kappa (κ—pain relief but can also associated with dysphoria), and nociceptin/orphanin FQ peptide receptor (NOP—involved in pain modulation, anxiety regulation, and memory processes) [3,4]. The intensity of the response elicited by these receptors can vary depending on ligand characteristics, which may act as agonists, partial agonists, or antagonists. However, most opioids used in clinical practice function as agonists, producing analgesic effects [5].

Commonly prescribed opioids include MOR, oxycodone (OXY), hydrocodone (HYD), tramadol (TRA), methadone (MET), buprenorphine (BUP), codeine (COD), and FEN (inpatient use) [6]. Illicit opioid use typically involves the recreational consumption of banned substances, such as heroin and illegally manufactured synthetic opioids like FEN, as well as the misuse of prescribed opioids. Notably, FEN, MOR, and OXY are frequently associated with preventable adverse drug reactions, abuse, and medication errors, underscoring the need for robust preventive measures and continued research to mitigate and reduce opioid-related harm [7]. Excessive or intentional misuse of opioids, whether consistent or sporadic, can lead to harmful physical or psychological outcomes, including adverse reactions and poisoning [8,9].

The intensity and symptoms of opioid poisoning depend on various factors, including the opioid’s potency, the administered dosage, the frequency of use, individual pharmacokinetics and pharmacodynamics, the presence of comorbidities, and potential interactions with other medications or substances [10]. Long-term use of opioids, typically defined as exceeding 60 days [11,12], often leads to pharmacological tolerance, requiring increasingly higher doses or a switch to a more potent opioid to achieve adequate pain suppression, thereby increasing the risk of poisoning and adverse events [13]. Chronic use of opioids is associated with physiological adaptations that result in tolerance and physical dependence [14].

According to Pergolizzi Jr et al. [15], 2.0% to 6.0% of patients who use prescribed opioids to treat chronic pain develop opioid use disorder. The American Medical Association also estimates that 3.0% to 19.0% of individuals who take prescription pain medications develop an addiction [15]. In 2021, among adults in the United States (USA) who used prescription opioids, 12.1% reported misuse, and 7.0% met criteria for prescription-related opioid use disorder [6]. In 2022, more than 130 million opioid prescriptions were dispensed, and nearly 8.5 million people reported having misused prescribed opioids [16]. The 2022 annual report from the Substance Abuse and Mental Health Services Administration [17] identified misuse of prescription pain relievers across age groups, with the highest prevalence observed among adults aged 26 and older (~7.0 million individuals).

The opioid crisis in the USA has escalated into a significant public health challenge, characterized by widespread misuse, addiction, and overdose deaths. Alarmingly, this pattern is not confined to the USA and is increasingly emerging as a global issue. Many countries are experiencing a rise in opioid-related problems, driven by similar factors, including the widespread availability of prescription opioids, weak regulatory frameworks, and limited public awareness of the risks of addiction [18]. Globally, the average opioid consumption has been increasing by 3.9% annually, rising from 27.5 morphine milligram equivalents per 1000 inhabitants per day (MME/1000/day) in 2015 to 29.5 in 2019 [19]. According to Ju et al. [19], the highest rates of opioid consumption in 2019 were observed in Canada (987.6 MME/1000/day), the USA (737.5 MME/1000/day), Australia (754.9 MME/1000/day), New Zealand (446.9 MME/1000/day), and several European countries (most notably, Switzerland—897.6 MME/1000/day, Germany—879.0 MME/1000/day, and Spain—863.1 MME/1000/day). The same study highlights a distinctive geographical pattern of opioid analgesic use that appears to be independent of both the Human Development Index and cancer prevalence rates [19]. Recent data on opioid use disorder indicate that, globally, the number of new cases, deaths, and disability-adjusted life years in 2021 increased by 49.25% compared with 1990 [20,21].

The opioids harmful effects included nausea, headache, sedation, constipation, pruritus, and sweating [22]. Other commonly observed effects were somnolence, dizziness, and vomiting, while potentially life-threatening events such as respiratory depression and cardiovascular complications [23]. The adverse effects and harms associated with opioid use, along with the increasing prescription rates, underscore the need for a thorough evaluation of the clinical consequences of these medications. Although the risks of opioid misuse and illicit consumption have been extensively studied, there is a notable gap in the literature regarding the harms arising from prescribed opioid use. The main novelty of this systematic review addresses this underexplored area by specifically assessing the adverse effects and toxicities associated with therapeutically indicated opioid use. This systematic review aims to evaluate the harmful (adverse and toxic) effects of prescribed opioids on the pediatric and the general population. Considering the physiological, metabolic, and pharmacokinetic differences between pediatric and general populations, it is also critical to investigate how these harmful effects may vary across the groups. Clarifying these patterns is essential to inform safer and more individualized opioid prescribing practices, ultimately improving clinical outcomes.

## 2. Materials and Methods

### 2.1. Protocol and Registration

The protocol for this systematic review was registered in the International Prospective Register of Systematic Reviews (PROSPERO; registration number CRD42023463794). All modifications to the initial protocol registered in PROSPERO were clearly reported and incorporated into the Methods section, in line with best practices for transparency and reproducibility in systematic reviews. These modifications are also detailed in the ‘additional information’ section of the PROSPERO record. The review was conducted and reported following the Preferred Reporting Items of Systematic Reviews and Meta-Analyses (PRISMA) guideline [24]. The complete PRISMA checklist is available in Tables S1 and S2 of the Supplementary Material. To formulate the guiding question, the PICO framework was applied as follows: P (Population) = pediatric and/or adult patients; I (intervention) = licit opioids use; C (comparison) = no control group; and O (outcome) = harmful effects of prescribed opioids (toxic and/or adverse effects).

### 2.2. Eligibility Criteria

Studies were included if they met the following criteria: (1) licit use of prescribed opioids; (2) reported and/or described harmful (toxic or adverse) effects of prescribed opioids; (3) full text published in English; and (4) data published from 2011 and 2024. Conversely, studies were excluded if they met any of the following criteria: (1) opioid use in the context of attempted suicide and/or *post-mortem* detection; (2) abuse or misuse of prescribed opioids; (3) full text not available or published in a non-English language; or (4) publication type classified as an editorial, conference paper, review, book, or a book chapter.

In this study, the term “harmful effects” refers to all cases of intoxication and/or adverse events resulting from the use of prescribed opioids. This includes unintended physiological or psychological effects arising from therapeutic use, accidental ingestion, or dosing errors. The aim is to standardize this terminology for clarity and to ensure a comprehensive analysis of the impact associated with prescription opioid use across diverse patient populations.

### 2.3. Search Strategy

Table S3 of the Supplementary Material presents the search strategy used to retrieve studies published between January 2011 to December 2024 from the electronic databases PubMed^®^, SciELO^®^, Web of Science^®^, and Embase^®^.

### 2.4. Study Selection and Data Extraction

The results of the systematic search from the databases were imported into Mendeley^®^ (Elsevier BV, Amsterdam, The Netherlands), and duplicate records were automatically removed. Three reviewers (L.S.L., M.E.A.G. and N.d.S.d.C.) independently screened the titles and abstracts to identify studies that met the eligibility criteria. Disagreements regarding study selection were resolved through discussion among the three reviewers, under the supervision of a fourth reviewer (C.S.O.). Records included after this screening phase were downloaded, and their full texts were assessed for relevance to the next stage of the review. Full-text articles that met all inclusion criteria had their data collected and synthesized qualitatively. Articles were excluded if they presented an incorrect outcome, study design, or setting, or if the full text was unavailable.

The data extraction was performed by two authors (L.S.L. and N.d.S.d.C.) and reviewed by a third author (C.S.O.). The synthesis method also includes the following information in descriptive tables: author, year, country, study period, number of participants, age (mean or median), sex, prescribed opioid, clinical symptoms, and type of exposure. The data were organized in an Excel^®^ 2016 spreadsheet (Microsoft^®^ Corp, Redmond, WA, USA).

### 2.5. Quality Assessment

Quality assessment was performed using the Risk Of Bias In Non-randomized Studies of Intervention (ROBINS-I) tool. This tool has seven domains, including bias due to confounding, bias in selection of participants into the study, bias in classification of interventions, bias due to deviations from intended interventions, bias due to missing data, bias in measurement of outcomes, bias in selection of the reported result which are classified into low risk, uncertain risk, and high risk of bias [25].

## 3. Results

### 3.1. Study Selection

**Figure 1:**
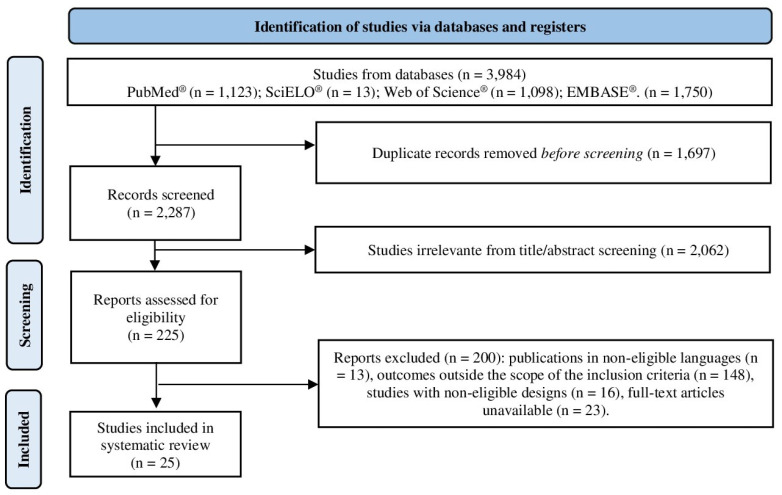
PRISMA flow diagram.

In this systematic review, 3984 scientific articles were retrieved from electronic databases. Of these, 1697 duplicate records were identified and removed, and the remaining 2287 were screened by title and abstract, resulting in 225 articles selected for full-text review. After full-text screening, 200 articles were excluded as they did not meet the eligibility criteria. Ultimately, 25 articles were included in the qualitative analysis (Figure 1).

### 3.2. Data Synthesis

We divided the data from the 25 selected articles into two distinct groups: (1) data from pediatric patients (0 to ≤18-year-old patients: 14 studies, Table S4 of Supplementary Material) and (2) data from the general population (11 studies, Table S5 of Supplementary Material). This grouping was done to facilitate comparisons in cases where the adult population was included, as none of the selected studies focused exclusively on adults. The age-based division followed classifications defined by the United States National Institutes of Health [26], the United Nations and its Children’s Fund [27], and the European Union [28], which collectively recognize patients under 18 years of age as children.

### 3.3. Study Characteristics

The majority of the systematically selected studies were conducted in Iran (60%), followed by the USA (28%), France (8%), and the United Kingdom (4%). In most cases of opioid-related harmful effects analyzed in this systematic review, the reported route of exposure was oral ingestion (Tables S4 and S5 of the Supplementary Material).

Although the majority of the studies on pediatric opioid-related harmful effects focused on MET (58%) and BUP (33%), the highest number of reported cases of harmful effects was associated with TRA (7441 cases), followed by BUP (2641 cases, including those combined with naloxone) (Table S4 of Supplementary Material). This suggests that, despite being less frequently studied, the harmful effects associated with TRA may be disproportionately represented in real-world pediatric settings. On the other hand, the majority of the opioid-related harmful effects on the general population focused on TRA (82% of the studies; 3014 cases) (Table S5 of Supplementary Material).

#### 3.3.1. Harmful Effects Caused by Prescribed Opioids in Pediatric Patients

**Figure 2:**
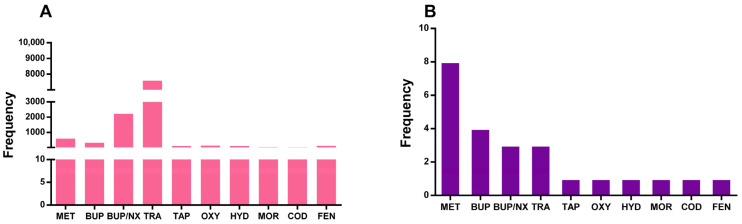
Number of patients (**A**) and studies (**B**) reporting opioid-related harmful effects on the pediatric population. MET—metadone; BUP—buprenorphine; BUP/NX—buprenorphine/naloxone; TRA—tramadol; TAP—tapentadol; OXY—oxycodone; HYD—hydrocodone; MOR—morphine; COD—codeine; FEN—fentanyl.

From the 25 studies systematically selected for qualitative analysis, 14 studies reported a pediatric group, from which 12 reported data on sex. From these data, we detected that females accounted for 52.9% of prescribed opioid harmful effects cases. The most frequently reported opioids prescribed for pediatric patients were MET (8 studies, 601 patients), BUP (4 studies, 321 patients), buprenorphine/naloxone (BUP/NX; 3 studies, 2225 patients), TRA (3 studies, 7586 patients), tapentadol (TAP) (1 study, 104 patients), OXY (1 study, 131 patients), HYD (1 study, 108 patients), MOR (1 study, 47 patients), COD (1 study, 40 patients), and FEN (1 study, 19 patients) (Figure 2A,B). Additionally, six studies reported cases of harmful effects involving more than one opioid, and three studies did not specify the opioid drug.

The data presented in Table 1 describe the percentage of prescribed opioids in the pediatric population, based on data from the systematically selected studies. In pediatric patients, TRA was the most prescribed opioid, accounting for 66.9% prescriptions; followed by BUP/NX tablets (18.90%), MET (3.90%), BUP (2.88%), BUP/NX film (1.06%), HYD (0.97%), MOR (0.42%), and COD (0.35%).

The percentage of most observed harmful effects in pediatric patients is reported in Table 2. This value corresponds to the percentage of occurrences of the sign within the total study population. The CNS was the primary target affected by the harmful effects reported in the evaluated studies, which included lethargy and drowsiness (650 patients, 4.70%), respiratory depression (456 patients, 3.30%), lethargy (459 patients, 3.30%), drowsiness (201 patients, 1.40%), and seizures (46 patients, 0.30%). Harmful effects on the gastrointestinal tract included vomiting and nausea (581 patients, 4.20%). Cardiac signs and/or symptoms reported were tachycardia (120 patients, 0.80%) and hypotension (65 patients, 0.40%). Psychiatric symptoms included irritability or agitation (172 patients, 1.30%) and confusion (73 patients, 0.50%). The only reported disturbance in the skin was itching/pruritus (97 patients, 0.70%).

#### 3.3.2. Harmful Effects Caused by Prescribed Opioids in the General Population

**Figure 3:**
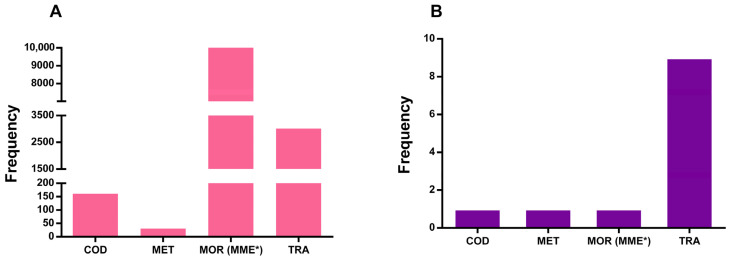
Number of patients (**A**) and studies (**B**) that reported opioid harmful effects in the general population. MOR (MME*) indicates MOR plus other opioids that were calculated in morphine equivalent dose. COD—codeine; MET—metadone; MOR—morphine; TRA—tramadol.

In studies analyzing effects caused by prescribed opioids in the general population (11 studies), females were reported to experience a higher rate of harmful effects (58.2%) compared to males. TRA was the most prescribed opioid associated with harmful effects, appearing in nine studies involving 3014 patients. Additionally, one study analyzed the use of multiple prescribed opioids, whose doses were standardized in MME, covering a large cohort of 98,140 patients. COD was reported in one study involving 161 patients, and MET in another study involving 31 patients (Figure 3). Notably, some studies included patients using more than one opioid, contributing to a broader understanding of the harmful effects of different medications when taken together by the same patient.

The data presented in Table 1 shows the percentage of prescribed opioid use, based on follow-up periods spanning from 2000 to 2020 and documented in articles published between 2011 and 2024. The most prescribed opioid in the general population was MOR (including MOR and other opioids calculated in MME), 96.76% prescriptions, followed by TRA (2.97%), COD (0.15%), and MET (0.03%).

Data on the number of patients associated with each reported harmful effect were found in nearly half of the analyzed studies. The description of harmful effects associated with opioid use in the general population is reported in Table 2. The most common cardiovascular effect reported was sinus bradycardia, affecting 536 patients (0.52%). Gastrointestinal effects, such as vomiting and nausea, were reported in 375 patients (0.36%). Seizures were observed in 630 patients (0.62%), and confusion was reported in 31 patients (0.03%). Respiratory, thoracic, and mediastinal disturbances were less common, as aspiration pneumonia and hyperventilation were detected in less than 1% of the sample.

### 3.4. Risk of Bias

The risk of bias in the included studies was assessed using the ROBINS-I tool, and the results are shown in Figure S1 of the Supplementary Material. Most studies (64%, 16 studies) were classified as having a moderate risk of bias. Bias due to missing data was uncertain in 52% of the studies, while bias due to participant selection was high in 16% and uncertain in 28% of the studies. Bias in measuring outcomes was uncertain in 40% of the studies, and bias in the selection of reported results was uncertain in 24% of the studies. These rates likely reflect the fact that many studies were retrospective, which does not guarantee that all patients having harmful effects due to prescribed opioids exposure were captured in the databases used in the studies, nor that all medical data were collected and reported.

## 4. Discussion

The opioid crisis emerged in the 1980s and 1990s with the production and widespread distribution of more potent opioids, often without adequate consideration of their addictive potential, adverse effects, or associated public health risks [29]. In this study, we show differences in the intensity and types of effects caused by prescribed opioids. Here, we detected a more pronounced variety of effects in pediatric patients compared to the general population. Additionally, we found differences between these groups regarding the prescribed opioid: MET, a long-acting opioid, was more prescribed for pediatric patients, while TRA was more frequently prescribed when the pediatric data were combined in the general population.

In our study, the majority of the information on the harmful effects of prescribed opioids comes from studies conducted in the USA and Iran. This fact reflects both the volume of research produced in these countries and their longstanding, focused efforts to address this critical public health issue. The concentration of studies in countries facing an opioid crisis likely stems from the widespread use of these medications and the resulting consequences. The prominence of the USA and Iran in the literature may be attributed to factors such as robust adverse event reporting systems, well-established research infrastructure, and the high incidence of opioid-related harms within their populations [30,31]. The patterns are further driven by factors such as high consumption rates and growing awareness of prescription drug-related harm risks [32,33].

Data from 2019 show that approximately 80% of the 600,000 drug-related deaths worldwide were linked to opioid use, which includes ~125,000 deaths (25%) resulting from opioid overdose [34]. Non-fatal overdoses are more common than fatal ones, as demonstrated by the great number of cases that do not result in death. Women are more likely than men to be diagnosed with chronic pain and to receive opioid prescriptions [35]. Biological, psychological, and cultural factors may contribute to this disparity [36]. In a study conducted at the Pharmacovigilance Centre Lareb between 2003 and 2021, Gustafsson et al. [8] reported 8769 cases of adverse drug reactions associated with opioid use in the Netherlands, with a high frequency observed in women (Reporting Odds Ratio female/male: 2.17). Similarly, Khodneva et al. [37] found that women were more likely to use prescription opioids and to experience cardiovascular side effects, suggesting sex-specific vulnerabilities. Accordingly, in our review, when sex was specified, women were more frequently prescribed opioids.

A study on *post-mortem* opioid overdose found that fatal aspiration was more common in young patients [38]. This may be attributed to the great experience and pharmacological tolerability to opioids in older patients, which can reduce the severity of symptoms such as vomiting and aspiration. In contrast, in the same study, younger patients with low tolerability levels appear more susceptible to these complications. Interestingly, higher MOR concentrations (0.50–2.0 μg/mL) were detected in the blood of deceased patients over 20 years of age, whereas those under 20 exhibited lower concentrations (<0.50 μg/mL). This comparison suggests that age-related differences, possibly due to the development of tolerability over time, may attenuate opioid-related toxicity. Age, therefore, plays a significant role in pharmacological tolerability, likely due to prolonged treatment duration and more frequent opioid exposure in older patients [38]. Consequently, a patient’s age may influence the type and severity of symptoms that manifest following opioid use. This review also observed that pediatric patients exhibited a wider range of harmful effects across multiple organ systems compared to those reported in studies involving the general population. This difference may be explained by lower tolerability to opioids in pediatric patients, making them more vulnerable to harmful effects than adults.

In this systematic review, we observed that pediatric patients were notably more susceptible to respiratory depression, indicating a heightened vulnerability to opioid-induced hypoventilation and oxygen desaturation. In contrast, adults exhibited a higher prevalence of seizures, possibly due to differences in drug metabolism or the more frequent use of TRA, which is known to increase seizure risk. Although miosis is a classical clinical sign of opioid intoxication, its frequent and expected occurrence might have led some studies to omit reporting it, potentially resulting in an underestimation of the overall opioid-related harmful effects, miosis was most commonly reported in pediatric cases; however, it was nearly absent in adults, potentially reflecting age-related variations in the autonomic nervous system and other responses to opioids [39]. Opioids impacted cardiovascular function in both groups, but effects were more severe in adults, likely due to metabolic variability and differences in the type of opioid prescribed. Agitation and irritability were more frequently reported in pediatric patients, suggesting that opioid toxicity in children may initially present with paradoxical agitation before progressing to CNS depression.

In the cardiovascular system, opioids strongly affect the central and peripheral nervous systems to regulate blood pressure and heart rate [40]. Opioid use is associated with an increased risk of cardiovascular disease, and opioid use may also be associated with myocardial fibrosis, which is linked to heart failure [41]. Furthermore, chronic use of MET and OXY has been associated with prolonged QT intervals [37]. Adult patients have a higher chance of having increased cardiovascular risk and cardiovascular comorbidities. Moreover, this age group typically requires increased doses and usage due to tolerance sometimes developed due to chronic use of these medications [13,38]. Therefore, a combination of these factors could explain the higher prevalence of cardiac disturbances in the general population when compared to the pediatric group.

Despite these differences, both groups shared common opioid harmful effects such as vomiting, drowsiness, and hypotension. Harmful effects caused by prescribed opioids require careful consideration when treating pediatric patients, as children may be more vulnerable than adults due to developmental changes in the brain’s reward and habit-formation centers. These changes may contribute to an increased risk of opioid misuse and the development of opioid use disorder later in life [42].

In this review, the primary administration route used by patients for both groups reporting opioid harmful effects was oral ingestion; however, accidental ingestion was more frequently reported in pediatric cases, highlighting a vulnerability specific to children. Our findings underscore the importance of systematically evaluating the adverse effects associated with prescribed opioids, particularly in pediatric patients. A careful assessment of the risk–benefit ratio is essential, especially in children’s care, to determine the proper use of opioids and to minimize potential harms to the patient.

## 5. Conclusions and Limitations

Metadone, buprenorphine, and buprenorphine/naloxone were the most frequently reported opioids among pediatric patients, often linked to respiratory depression and neurological disturbances. In contrast, tramadol, more commonly prescribed to the general population, was strongly associated with seizures and cardiovascular complications. The comparison between groups reveals key differences: children were more vulnerable to respiratory and neurological effects, while adults experienced more cardiovascular harm. These findings underscore the need for tailored prescribing practices and careful monitoring, particularly in vulnerable populations such as children, to reduce the risk of serious adverse effects while ensuring effective pain management.

Certain limitations were identified in our assessment of this issue, we detected a few studies specifically included in the hypothesis and/or major outcomes of the harmful effects of prescribed opioids, even though many report on their use. Research access on opioid-related harms, particularly in therapeutic contexts, is limited, and few studies have focused on intoxication symptoms, which was the central aim of this review. Group division enabled comparison between the pediatric versus the general population. However, this experimental design introduced potential bias, especially as some children may have been included in the general population group, since several studies did not discriminate the harmful effects by age. Most studies focused on young adults; only one included patients with a mean age over 60, revealing a lack of data on older adults. Additionally, no studies exclusively involving adult patients met our inclusion criteria. Several reporting systems only classify cases as ‘intoxication’ without describing specific harmful effects, limiting retrospective analysis and pharmacovigilance. Most selected studies originated from just two countries (the USA and Iran), with only a few from France and the UK, which restricts generalizability to other countries. Despite the opioid epidemic highlighting the need for improved pain management, limited data on the adverse effects of prescribed opioids remain a barrier to a better understanding of opioid toxicology and to safer opioid prescribing.

## Figures and Tables

**Table 1:** Percentage of prescribed opioid drugs in the systematically selected articles published between 2011 and 2024.

Prescribed Opioid (%)	Pediatric Patients	General Population
BUP	2.86	-
BUP/NX	0.83	-
BUP/NX film	1.06	-
BUP/NX tablets	18.43	-
COD	0.35	0.16
FEN	0.17	-
HYD	0.97	-
MET	3.92	0.03
MOR	0.42	-
MOR (MME) *	-	96.76
Other opioids	2.20	-
OXY	1.17	-
TAP	0.90	-
TRA	66.35	2.30

* MOR plus other opioids calculated in morphine equivalent dose (MME).

**Table 2:** Percentage of patients who present harmful effects * of prescribed opioids in the systematically selected articles published between 2011 and 2024.

Harmful Effect	Pediatric Patients (%)	General Population (%)
*Gastrointestinal*	4.43	0.48
Nausea and vomiting	4.22	0.36
*Cardiac*	3.46	1.18
Hypotension	0.47	0.01
Dominant S wave	-	0.38
Long QTc interval	-	0.25
Right axis deviation		0.33
Sinus bradycardia	-	0.52
Tachycardia	0.87	0.005
*Psychiatric*	1.80	0.04
Agitation	1.30	-
Confusion	0.53	0.03
*Nervous system*	18.87	1.22
Cyanosis	0.48	-
Bradypnea	1.03	-
Dizziness	0.22	0.34
Drowsiness	1.46	0.07
Drowsiness/lethargy	4.72	-
Headache	-	0.05
Lethargy	3.33	-
Respiratory depression	3.32	-
Seizure	0.33	0.62
*Respiratory, thoracic, and mediastinal*	2.43	0.01
Aspiration pneumonia	0.01	0.004
Variation in oxygen saturation level	0.60	-
*Ophtamological*	4.64	0.01
Blurred vision	-	0.01
Miosis **	4.49	-
*Other signs and administration site conditions*	1.50	0.11
Hyperhidrosis	-	0.04
Itching/pruritus	0.70	0.03

* Only the harmful effects with the highest percentages were described. ** Opioid intoxication sing.

## Data Availability

No new data were created or analyzed in this study.

## Supplementary Materials

The following supporting information can be downloaded at: https://www.mdpi.com/article/10.3390/ph18101429/s1. Table S1: PRISMA 2020 abstract checklist. Table S2: PRISMA 2020 checklist. Table S3: Search strategy for each electronic database; Table S4: Summary of systematically selected studies from 2011 to 2024 that evaluated the harmful effects of opioids in pediatric patients (Group 1); Table S5: Summary of systematically selected studies from 2011 to 2024 that evaluated the harmful effects of opioids in the general population (Group 2). Figure S1: Risk of bias of the 25 selected articles. References [43,44,45,46,47,48,49,50,51,52,53,54,55,56,57,58,59,60,61,62,63,64,65,66,67] are cited in the Supplementary Materials.

## References

[1] Carli M., Donnini S., Pellegrini C., Coppi E., Bocci G. (2020). Opioid receptors beyond pain control: The role in cancer pathology and the debated importance of their pharmacological modulation. Pharmacol. Res..

[2] Machelska H., Celik M.Ö. (2020). Opioid receptors in immune and glial cells—Implications for pain control. Front. Immunol..

[3] Stein C. (2016). Opioid receptors. Annu. Rev. Med..

[4] Wang Y., Zhuang Y., DiBerto J.F., Zhou X.E., Schmitz G.P., Yuan Q., Jain M.K., Liu W., Melcher K., Jiang Y. (2023). Structures of the entire human opioid receptor family. Cell.

[5] James A., Williams J. (2020). Basic opioid pharmacology—An update. Br. J. Pain.

[6] Han B., Jones C.M., Einstein E.B., Dowell D., Compton W.M. (2024). Prescription opioid use disorder among adults reporting prescription opioid use with or without misuse in the United States. J. Clin. Psychiatry.

[7] Michienzi A.E., Borek H.A. (2022). Emerging agents of substance use/misuse. Emerg. Med. Clin. N. Am..

[8] Gustafsson M., Matos C., Joaquim J., Scholl J., van Hunsel F. (2023). Adverse drug reactions to opioids: A study in a national pharmacovigilance database. Drug Saf..

[9] Gustafsson M., Silva V., Valeiro C., Joaquim J., van Hunsel F., Matos C. (2024). Misuse, abuse and medication errors’ adverse events associated with opioids—A systematic review. Pharmaceuticals.

[10] Albores-Garcia D., Cruz S.L. (2023). Fentanyl and other new psychoactive synthetic opioids. Challenges to prevention and treatment. Rev. Investig. Clin. Org. Hosp. Enfermedades Nutr..

[11] Mohamadi A., Chan J.J., Lian J., Wright C.L., Marin A.M., Rodriguez E.K., von Keudell A., Nazarian A. (2018). Risk factors and pooled rate of prolonged opioid use following trauma or surgery: A systematic review and meta-(regression) analysis. J. Bone Jt. Surg. Am..

[12] Hurtado I., Robles C., García-Sempere A., Llopis-Cardona F., Sánchez-Sáez F., Rodríguez-Bernal C., Peiró S., Sanfélix-Gimeno G. (2024). Long-term use of prescription opioids for non-cancer pain and mortality: A population-based, propensity-weighted cohort study. Public Health.

[13] Basaran M.B., Koca R.O., Gormus Z.I.S. (2024). Therapeutic innovations against opioid tolerance and addiction. Curr. Behav. Neurosci. Rep..

[14] Volkow N.D., Blanco C. (2021). The changing opioid crisis: Development, challenges and opportunities. Mol. Psychiatry.

[15] Pergolizzi J.V., Raffa R.B., Rosenblatt M.H. (2020). Opioid withdrawal symptoms, a consequence of chronic opioid use and opioid use disorder: Current understanding and approaches to management. J. Clin. Pharm. Ther..

[16] Centers for Disease Control and Prevention (CDC) (2024). About Prescription Opioids. https://www.cdc.gov/overdose-prevention/about/prescription-opioids.html.

[17] Substance Abuse and Mental Health Services Administration (SAMHSA) (2023). Key Substance Use and Mental Health Indicators in the United States: Results from the 2022 National Survey on Drug Use and Health. https://www.samhsa.gov/data/report/2022-nsduh-annual-national-report.

[18] Robert M., Jouanjus E., Khouri C., Fouilhé Sam-Laï N., Revol B. (2023). The opioid epidemic: A worldwide exploratory study using the WHO pharmacovigilance database. Addiction.

[19] Ju C., Wei L., Man K.K.C., Wang Z., Ma T.-T., Chan A.Y.L., Brauer R., Chui C.S.L., Chan E.W., Jani Y.H. (2022). Global, regional, and national trends in opioid analgesic consumption from 2015 to 2019: A longitudinal study. Lancet Public Health.

[20] Fang M., Zhang Q., Peng J., Yao W., Feng W., Wan X. (2025). Global, regional, and national burden of opioid use disorder from 1990 to 2021: A statistical analysis of incidence, mortality, and disability-adjusted life years. BMC Public Health.

[21] Han F., Liu B., Wang L., Zhu S., Li X., Kang S., Niu X., Song J., Wu Y. (2025). Global, Regional, and National Epidemiology of Opioid Use Disorder Among Adolescents and Young Adults, 1990–2019. J. Adolesc. Health.

[22] Meyer M., Strazdins E., Guessoum A., Westenberg J.N., Appenzeller-Herzog C., Cattaneo M.E.G.V., Krausz R.M., Dürsteler K.M., Lang U.E., Hemkens L.G. (2025). Relative risks of adverse effects across different opioid agonist treatments—A systematic review and meta-analysis. Addiction.

[23] Zimmerman A., Laitman A. (2024). Safe management of adverse effects associated with prescription opioids in the palliative care population: A narrative review. J. Clin. Med..

[24] Page M.J., McKenzie J.E., Bossuyt P.M., Boutron I., Hoffmann T.C., Mulrow C.D., Shamseer L., Tetzlaff J.M., Akl E.A., Brennan S.E. (2021). The PRISMA 2020 statement: An updated guideline for reporting systematic reviews. BMJ.

[25] Sterne J.A.C., Hernán M.A., Reeves B.C., Savović J., Berkman N.D., Viswanathan M., Henry D., Altman D.G., Ansari M.T., Boutron I. (2016). ROBINS-I: A tool for assessing risk of bias in non-randomised studies of interventions. BMJ.

[26] United States National Institutes of Health (NIH) (2019). Guidelines for the Review of Inclusion on the Basis of Sex/Gender, Race, Ethnicity, and Age in Clinical Research. https://grants.nih.gov/grants/peer/guidelines_general/Review_Human_subjects_Inclusion.pdf.

[27] United Nations International Children’s Emergency Fund (UNICEF) (1989). Convention on the Rights of the Child. https://www.unicef.org/child-rights-convention/convention-text.

[28] European Commission (2022). EU Action on the Rights of the Child. https://ec.europa.eu/info/policies/justice-and-fundamental-rights/rights-child/eu-action-rights-child_en.

[29] Vadhel A., Bashir S., Mir A.H., Girdhar M., Kumar D., Kumar A., Mohan A., Malik T., Mohan A. (2023). Opium alkaloids, biosynthesis, pharmacology and association with cancer occurrence. Open Biol..

[30] Mohebbi E., Haghdoost A.A., Noroozi A., Vardanjani H.M., Hajebi A., Nikbakht R., Mehrabi M., Kermani A.J., Salemianpour M., Baneshi M.R. (2018). Awareness and attitude towards opioid and stimulant use and lifetime prevalence of the drugs: A study in 5 large cities of Iran. Int. J. Health Policy Manag..

[31] Chen Q., Sterner G., Rhubart D., Newton R., Shaw B., Scanlon D. (2024). Creating a robust coordinated data and policy framework for addressing substance use issues in the United States. Int. J. Drug Policy.

[32] Marks C., Carrasco-Escobar G., Carrasco-Hernández R., Johnson D., Ciccarone D., Strathdee S.A., Smith D., Bórquez A. (2021). Methodological approaches for the prediction of opioid use-related epidemics in the United States: A narrative review and cross-disciplinary call to action. Transl. Res..

[33] Smith N.Z.Y., Thornton J.D., Fenton S.H., Simmons D., Champagne-Langabeer T. (2023). Helpful, unnecessary, or harmful: A systematic review of the effects of prescription drug monitoring program use on opioid prescriptions. Pharmacoepidemiol. Drug Saf..

[34] Erkok S.D., Gallois R., Leegwater L., Gonzalez P.C., van Asten A., McCord B. (2024). Combining surface-enhanced Raman spectroscopy (SERS) and paper spray mass spectrometry (PS-MS) for illicit drug detection. Talanta.

[35] Muriel J., Barrachina J., Del Barco G., Carvajal C., Escorial M., Margarit C., Ballester P., Peiró A.M. (2023). Impact of CYP2D6 genotype on opioid use disorder deprescription: An observational prospective study in chronic pain with sex-differences. Front. Pharmacol..

[36] Templeton K.J. (2020). Sex and gender issues in pain management. J. Bone Jt. Surg. Am..

[37] Khodneva Y., Muntner P., Kertesz S., Kissela B., Safford M.M. (2016). Prescription opioid use and risk of coronary heart disease, stroke, and cardiovascular death among adults from a prospective cohort (REGARDS Study). Pain Med..

[38] Nicolakis J., Gmeiner G., Reiter C., Seltenhammer M.H. (2020). Aspiration in lethal drug abuse—A consequence of opioid intoxication. Int. J. Leg. Med..

[39] Takla M., Saadeh K., Tse G., Huang C.L.H., Jeevaratnam K. (2023). Ageing and the autonomic nervous system. Biochemistry and Cell Biology of Ageing: Part IV, Clinical Science.

[40] Chow S.L., Sasson C., Benjamin I.J., Califf R.M., Compton W.M., Oliva E.M., Robson C., Sanchez E.J. (2021). Opioid use and its relationship to cardiovascular disease and brain health: A presidential advisory from the American Heart Association. Circulation.

[41] Liew S.M., Chowdhury E.K., Ernst M.E., Gilmartin-Thomas J., Reid C.M., Tonkin A., Neumann J., McNeil J.J., Kaye D.M. (2022). Prescribed opioid use is associated with adverse cardiovascular outcomes in community-dwelling older persons. ESC Heart Fail..

[42] Yaster M., McNaull P.P., Davis P.J. (2020). The opioid epidemic in pediatrics: A 2020 update. Curr. Opin. Anaesthesiol..

[43] Pedapati E.V., Bateman S.T. (2011). Toddlers requiring pediatric intensive care unit admission following at-home exposure to buprenorphine/naloxone. Pediatr. Crit. Care Med..

[44] Lavonas E.J., Banner W., Bradt P., Bucher-Bartelson B., Brown K.R., Rajan P., Green J.L. (2013). Root causes, clinical effects, and outcomes of unintentional exposures to buprenorphine by young children. J. Pediatr..

[45] Jabbehdari S., Farnaghi F., Shariatmadari S.F., Jafari N., Mehregan F.F., Karimzadeh P. (2013). Accidental children poisoning with methadone: An Iranian pediatric sectional study. Iran. J. Child Neurol..

[46] Bazmamoun H., Fayyaz A., Khajeh A., Sabzehei M.K., Khezrian F. (2014). A study of methadone-poisoned children referred to Hamadan’s Besat Hospital/Iran. Iran. J. Child Neurol..

[47] Sharif M.R., Nouri S. (2015). Clinical signs and symptoms and laboratory findings of methadone poisoning in children. Iran. J. Pediatr..

[48] Borys D., Stanton M., Gummin D., Drott T. (2015). Tapentadol toxicity in children. Pediatrics.

[49] Hamedi A., Ataei A., Balali M.R., Ghahremani S., Ghahremani S. (2016). A cross-sectional study on pediatric methadone poisoning in northeast of Iran. Asia Pac. J. Med. Toxicol..

[50] Stassinos G.L., Gonzales L., Klein-Schwartz W. (2019). Characterizing the toxicity and dose-effect profile of tramadol ingestions in children. Pediatr. Emerg. Care.

[51] Toce M.S., Burns M.M., O’Donnell K.A. (2017). Clinical effects of unintentional pediatric buprenorphine exposures: Experience at a single tertiary care center. Clin. Toxicol..

[52] Carreiro S., Miller S., Wang B., Wax P., Campleman S., Manini A.F. (2019). Clinical predictors of adverse cardiovascular events for acute pediatric drug exposures. Clin. Toxicol..

[53] Riasi H., Rabiee N., Chahkandi T., Arzanin F., Atary S.K., Salehi F. (2021). Electrocardiographic changes in children with acute opioid poisoning: A cross-sectional study. Pediatr. Emerg. Care.

[54] Farnaghi F., Gholami N., Hassanian-Moghaddam H., McDonald R., Zamanzadeh R., Zamani N. (2021). Unintentional buprenorphine and methadone poisoning in children: A matched observational study. Clin. Toxicol..

[55] Cohen N., Mathew M., Davis A., Brent J., Wax P., Schuh S., ToxIC Pediatric Opioid Exposure Study Group (2022). Predictors of severe outcome following opioid intoxication in children. Clin. Toxicol..

[56] Caré W., Tangre A., Dufayet L., Lekens B., Laborde-Casterot H., Langrand J., Vodovar D. (2022). Exposure to immediate-release tramadol in children 6 years and under—A nationwide French poison control center study. Clin. Toxicol..

[57] Emamhadi M., Sanaei-Zadeh H., Nikniya M., Zamani N., Dart R.C. (2012). Electrocardiographic manifestations of tramadol toxicity with special reference to their ability for prediction of seizures. Am. J. Emerg. Med..

[58] Eizadi-Mood N., Ozcan D., Sabzghabaee A.M., Mirmoghtadaee P., Hedaiaty M. (2014). Does naloxone prevent seizure in tramadol intoxicated patients?. Int. J. Prev. Med..

[59] Asadi P., Kasmaei V.M., Ziabari S.Z., Zohrevandi B., Manesh A.M. (2015). Prevalence of tramadol consumption in first seizure patients; a one-year cross-sectional study. Emergency.

[60] Farzaneh E., Amani F., Etemad F. (2016). A clinico-epidemiologic study on patients with opium toxicity treated at Ardabil Hospitals, Iran, 2014–2015. Asia Pac. J. Med. Toxicol..

[61] Ghamsari A.A., Dadpour B., Najari F. (2016). Frequency of electrocardiographic abnormalities in tramadol poisoned patients: A brief report. Emergency.

[62] Moghadam P.H., Zarei N., Farsi D., Abbasi S., Mofidi M., Rezai M., Mahshidfar B. (2016). Electrocardiographic changes in patients with tramadol-induced idiosyncratic seizures. Turk. J. Emerg. Med..

[63] Ahmadimanesh M., Shadnia S., Rouini M.R., Sheikholeslami B., Ahsani Nasab S., Ghazi-Khansari M. (2018). Correlation between plasma concentrations of tramadol and its metabolites and the incidence of seizure in tramadol-intoxicated patients. Drug Metab. Pers. Ther..

[64] Bedson J., Chen Y., Ashworth J., Hayward R.A., Dunn K.M., Jordan K.P. (2019). Risk of adverse events in patients prescribed long-term opioids: A cohort study in the UK clinical practice research Datalink. Eur. J. Pain.

[65] Mohammadpour A., Ashkezari M.D., Farahmand B., Shokrzadeh M. (2019). Demographic characteristics and functional performance of the kidneys and hearts of patients with acute tramadol toxicity. Drug Res..

[66] Ahmadimanesh M., Naeini M.B., Rouini M.R., Shadnia S., Ghazi-Khansari M. (2020). Assessment of tramadol pharmacokinetics in correlation with CYP2D6 and clinical symptoms. Drug Metab. Pers. Ther..

[67] Caré W., Pinel S., Dufayet L., Langrand J., Micallef J., Vodovar D. (2023). Trends in adverse drug reactions related to oral weak opioid analgesics in therapeutic use in adults: A 10-year French vigilances retrospective study. Fundam. Clin. Pharmacol..

