# Supplementary material for: Harmful Effects of Prescribed Opioids in Children and Adults: A Systematic Review
Source: Pharmaceuticals (Basel). 2025 Sep 24;18(10):1429. doi: 10.3390/ph18101429 (PMC12567551; doi:10.3390/ph18101429)
Supplement: Supplementary file 1 [file pharmaceuticals-18-01429-s001.zip › pharmaceuticals-3803612-supplementary.pdf]

SUPPLEMENTARY MATERIAL

Harmful Effects of Prescribed Opioids: A Systematic Review with a Focus on Pediatric Patients and the General Population

Luíza S. Lima[1,2], Nayara de S. da Costa[1,2], Maria Eduarda A. Galiciolli[1,2] , Quelen I. Garlet[3], João José Joaquim[4], Cláudia S. Oliveira[1,2 *], Cristiano Matos[4 *]

[1] Instituto de Pesquisa Pelé Pequeno Príncipe, Curitiba 80250-060, Brazil; luizaelima04@gmail.com (L.S.L.); costasouzanayara@gmail.com (N.d.S.d.C.); dudagaliciolli@hotmail.com (M.E.A.G.)

2 Faculdades Pequeno Príncipe, Curitiba 80230-020, Brazil;

3 Universidade Federal do Paraná, Programa de Pós-Graduação em Farmacologia, Curitiba – Centro Politécnico, Curitiba, 800615300-000, Brazil; quelen.garlet@ufpr.br

4 ESTESC-Coimbra Health School, Instituto Politécnico De Coimbra, Farmácia, 3045-043 Coimbra, Portugal; jjj@estesc.ipc.pt (J.J.J)

*Corresponding author:

Cristiano Matos. (cristiano.matos@estesc.ipc.pt); Claúdia de Oliveira (claudia.sirlene@professor.fpp.edu.br).

Table S1 of supplementary material: PRISMA 2020 abstract checklist

Section and Topic	Item #	Checklist item	Reported (Yes/No)
**TITLE**			
Title	1	Identify the report as a systematic review.	Page 1, lines 2-3
**BACKGROUND**			
Objectives	2	Provide an explicit statement of the main objective(s) or question(s) the review addresses.	Page 1, lines 19-21
**METHODS**			
Eligibility criteria	3	Specify the inclusion and exclusion criteria for the review.	Page 1, lines 24-26.
Information sources	4	Specify the information sources (e.g. databases, registers) used to identify studies and the date when each was last searched.	Page 1, lines 21-24
Risk of bias	5	Specify the methods used to assess risk of bias in the included studies.	Page 1, line 29
Synthesis of results	6	Specify the methods used to present and synthesise results.	Page 1, lines 26-29
**RESULTS**			
Included studies	7	Give the total number of included studies and participants and summarise relevant characteristics of studies.	Page 1, lines 29-31.
Synthesis of results	8	Present results for main outcomes, preferably indicating the number of included studies and participants for each. If meta-analysis was done, report the summary estimate and confidence/credible interval. If comparing groups, indicate the direction of the effect (i.e. which group is favoured).	Page 1, lines 29 - 35
**DISCUSSION**			
Limitations of evidence	9	Provide a brief summary of the limitations of the evidence included in the review (e.g. study risk of bias, inconsistency and imprecision).	Page 1, line 35
Interpretation	10	Provide a general interpretation of the results and important implications.	Page 1, line 35 - 39
**OTHER**			
Funding	11	Specify the primary source of funding for the review.	Not applicable.
Registration	12	Provide the register name and registration number.	Not applicable.

Table S2 of supplementary material: PRISMA 2020 checklist

Section and Topic	Item #	Checklist item	Location where item is reported
**TITLE**			
Title	1	Identify the report as a systematic review.	Page 1, Line 3
**ABSTRACT**			
Abstract	2	See the PRISMA 2020 for Abstracts checklist.	Page 1, lines 16-39
**INTRODUCTION**			
Rationale	3	Describe the rationale for the review in the context of existing knowledge.	Page 2-4, lines 47 - 117
Objectives	4	Provide an explicit statement of the objective(s) or question(s) the review addresses.	Page 4, lines 119-125
**METHODS**			
Eligibility criteria	5	Specify the inclusion and exclusion criteria for the review and how studies were grouped for the syntheses.	Page 4, lines 142-155
Information sources	6	Specify all databases, registers, websites, organisations, reference lists and other sources searched or consulted to identify studies. Specify the date when each source was last searched or consulted.	Page 4, lines 158 - 160
Search strategy	7	Present the full search strategies for all databases, registers and websites, including any filters and limits used.	Table S3 of supplementary information
Selection process	8	Specify the methods used to decide whether a study met the inclusion criteria of the review, including how many reviewers screened each record and each report retrieved, whether they worked independently, and if applicable, details of automation tools used in the process.	Page 4 – 5, lines 157 - 167
Data collection process	9	Specify the methods used to collect data from reports, including how many reviewers collected data from each report, whether they worked independently, any processes for obtaining or confirming data from study investigators, and if applicable, details of automation tools used in the process.	Page 5, lines 163 -174
Data items	10a	List and define all outcomes for which data were sought. Specify whether all results that were compatible with each outcome domain in each study were sought (e.g. for all measures, time points, analyses), and if not, the methods used to decide which results to collect.	Page 5, lines 174 -177
	10b	List and define all other variables for which data were sought (e.g. participant and intervention characteristics, funding sources). Describe any assumptions made about any missing or unclear information.	Page 5, lines 174 -177
Study risk of bias assessment	11	Specify the methods used to assess risk of bias in the included studies, including details of the tool(s) used, how many reviewers assessed each study and whether they worked independently, and if applicable, details of automation tools used in the process.	Page 5, lines 180 - 185

Section and Topic	Item #	Checklist item	Location where item is reported
Effect measures	12	Specify for each outcome the effect measure(s) (e.g. risk ratio, mean difference) used in the synthesis or presentation of results.	Page 5, line 176
Synthesis methods	13a	Describe the processes used to decide which studies were eligible for each synthesis (e.g. tabulating the study intervention characteristics and comparing against the planned groups for each synthesis (item #5)).	Out of the scope of this systematic review
	13b	Describe any methods required to prepare the data for presentation or synthesis, such as handling of missing summary statistics, or data conversions.	Out of the scope of this systematic review
	13c	Describe any methods used to tabulate or visually display results of individual studies and syntheses.	Page 5, lines 174 -177
	13d	Describe any methods used to synthesize results and provide a rationale for the choice(s). If meta-analysis was performed, describe the model(s), method(s) to identify the presence and extent of statistical heterogeneity, and software package(s) used.	Out of the scope of this systematic
	13e	Describe any methods used to explore possible causes of heterogeneity among study results (e.g. subgroup analysis, meta-regression).	Out of the scope of this systematic
	13f	Describe any sensitivity analyses conducted to assess robustness of the synthesized results.	Out of the scope of this systematic
Reporting bias assessment	14	Describe any methods used to assess risk of bias due to missing results in a synthesis (arising from reporting biases).	Out of the scope of this systematic
Certainty assessment	15	Describe any methods used to assess certainty (or confidence) in the body of evidence for an outcome.	Out of the scope of this systematic
**RESULTS**			
Study selection	16a	Describe the results of the search and selection process, from the number of records identified in the search to the number of studies included in the review, ideally using a flow diagram.	Page 5, lines 189 – 194; Figure 1.
	16b	Cite studies that might appear to meet the inclusion criteria, but which were excluded, and explain why they were excluded.	Out of the

Section and Topic	Item #	Checklist item	Location where item is reported
			scope of this systematic
Study characteristics	17	Cite each included study and present its characteristics.	Tables S4 and S5 of the supplementary material
Risk of bias in studies	18	Present assessments of risk of bias for each included study.	Figure S1 of the Supplementary Material
Results of individual studies	19	For all outcomes, present, for each study: (a) summary statistics for each group (where appropriate) and (b) an effect estimate and its precision (e.g. confidence/credible interval), ideally using structured tables or plots.	Tables S4 and S5 of the supplementary material
Results of syntheses	20a	For each synthesis, briefly summarise the characteristics and risk of bias among contributing studies.	Pages 6 - 9, lines 299 – 304.
	20b	Present results of all statistical syntheses conducted. If meta-analysis was done, present for each the summary estimate and its precision (e.g. confidence/credible interval) and measures of statistical heterogeneity. If comparing groups, describe the direction of the effect.	Out of the scope of this systematic
	20c	Present results of all investigations of possible causes of heterogeneity among study results.	Out of the scope of this systematic
	20d	Present results of all sensitivity analyses conducted to assess the robustness of the synthesized results.	Out of the scope of this systematic
Reporting biases	21	Present assessments of risk of bias due to missing results (arising from reporting biases) for each synthesis assessed.	Out of the scope of this systematic
Certainty of evidence	22	Present assessments of certainty (or confidence) in the body of evidence for each outcome assessed.	Out of the scope of this systematic
**DISCUSSION**			

Section and Topic	Item #	Checklist item	Location where item is reported
Discussion	23a	Provide a general interpretation of the results in the context of other evidence.	Pages 9 -11 , lines 306 – 391.
	23b	Discuss any limitations of the evidence included in the review.	Pages 11 - 12, lines 403 – 420.
	23c	Discuss any limitations of the review processes used.	Pages 11 - 12, lines 403 – 420.
	23d	Discuss implications of the results for practice, policy, and future research.	Page 11, line 394 – 402.
**OTHER INFORMATION**			
Registration and protocol	24a	Provide registration information for the review, including register name and registration number, or state that the review was not registered.	Page 4 lines 128-133
	24b	Indicate where the review protocol can be accessed, or state that a protocol was not prepared.	Page 4 line 128-133
	24c	Describe and explain any amendments to information provided at registration or in the protocol.	Page for, lines 130-133
Support	25	Describe sources of financial or non-financial support for the review, and the role of the funders or sponsors in the review.	None.
Competing interests	26	Declare any competing interests of review authors.	None.
Availability of data, code and other materials	27	Report which of the following are publicly available and where they can be found: template data collection forms; data extracted from included studies; data used for all analyses; analytic code; any other materials used in the review.	Supplementar y material

Table S3 of supplementary material: Search strategy for each electronic database.

Electronic database	Search strategy
PubMed® (title/abstract)	(“opioids” OR “opiates” OR “oxycodone” OR “morphine” OR “tramadol” OR “codeine” OR “fentanyl”) AND (“intoxication” OR “poisoning”) NOT (“rats” OR “mouse” OR “mice” OR “rodent”)
SciELO® (title/abstract)	
Web of Science® (title/abstract/keywords)	
EMBASE® (title/abstract/keywords)	

Table S4 of supplementary material. Summary of systematically selected studies from 2011 to 2024 that evaluated the harmful effects of opioids in pediatric patients (Group 1).

Reference	Country	Follow up	Sample characteristics			Harmful effects	
			Sex [N (%)]	Age (years)	Drugs [N (%)]	Clinical symptoms [N (%)]	Type of exposure
Pedapati and Bateman 2011 [43]	USA	2007-2009	Female + Male: 9 (100.0)	< 3	Buprenorphine/naloxone: 9 (100.0)	Drowsiness or lethargy: 9 (100.0)<br>Miosis: 6 (67.0)<br>Decline in respiratory function: 5 (56.0)<br>Vomiting: 4 (44.0)<br>Agitation or irritability: 3 (33.0)<br>Confusion: 3 (33.0)<br>Diaphoresis: 2 (22.0)<br>Hypoxia: 1 (11.0)	*Per oral* (ingestion)
Lavonas et al. 2013 [44]	USA	2009-2012	Total buprenorphine<br>Female: 1127 (47.4)<br>Male: 1229 (51.6)<br><br>Buprenorphine tablets<br>Female: 79 (51.3)<br>Male: 74 (48.1)<br><br>Buprenorphine/naloxone tablets<br>Female: 983 (46.7)<br>Male: 1106 (52.5)<br><br>Buprenorphine/naloxone film<br>Female: 64 (54.2)<br>Male: 49 (41.5)	0 - < 6	Buprenorphine tablets: 154 (6.5)<br>Buprenorphine/naloxone tablets: 2107 (88.5)<br>Buprenorphine/naloxone film: 118 (5.0)	**Buprenorphine tablets**<br>Lethargy: 36 (23.0)<br>Respiratory depression: 17 (11.0)<br>Miosis: 10 (6.0)<br>Tachycardia: 9 (6.0)<br>Vomiting: 7 (4.0)<br>Agitation: 6 (4.0)<br>Hypotension: 6 (4.0)<br>Ataxia: 5 (3.0)<br>Irritability: 5 (3.0)<br>Bradycardia: 2 (2.0)<br>Pallor: 2 (2.0)<br>Pyrexia: 2 (2.0)<br>Arrhythmia: 1 (1.0)<br>Cardiac arrest: 1 (1.0)<br>Cyanosis: 1 (1.0)<br>Brain edema: 1 (1.0)<br>Bronchospasm: 1 (1.0)<br>Death: 1 (1.0)<br>Dizziness: 1 (1.0)	Sublingual gaze palsy formulation

Eyelid ptosis: 1 (1.0)
Hypothermia: 1 (1.0)
Increased bronchial secretion: 1(1.0)
Myocardial infarction: 1 (1.0)
Nystagmus: 1 (1.0)
Pupil fixed: 1 (1.0)
Unresponsive to stimuli: 1 (1.0)
Pneumonia aspiration: 1 (1.0)
Respiratory arrest: 1 (1.0)
Stridor: 1 (1.0)
Hypertension: 1 (1.0)
Hemorrhage: 1 (1.0)

Buprenorphine/naloxone tablets
Lethargy: 375 (18)
Respiratory depression: 200 (9.5)
Miosis: 172 (8.0)
Vomiting: 134 (6.0)
Agitation: 62 (3.0)
Tachycardia: 51 (2.0)
Bradycardia: 42 (2.0)
Ataxia: 38 (1.8)
Hypotension: 37 (1.7)
Irritability: 33 (1.6)
Somnolence: 28 (1.3)
Cyanosis: 15 (0.7)
Pyrexia: 11 (0.5)
Hypertension: 9 (0.4)
Confusional state: 8 (0.4)
Dizziness: 6 (0.3)
Depressed level of consciousness: 6 (0.3)
Flushing: 6 (0.3)
Pallor: 6 (0.3)
Wheezing: 6 (0.3)

	Dysarthria: 5 (0.2)<br>Coma: 4 (0.2)<br>Nausea: 4 (0.2)<br>Respiratory arrest: 4 (0.2)<br>Stridor: 4 (0.2)<br>Abnormal behavior: 3 (0.14)<br>Dyspnea: 3 (0.14)<br>Gastrointestinal sounds abnormal: 3 (0.2)<br>Hallucination: 3 (0.14)<br>Hypoxia: 3 (0.14)<br>Loss of consciousness: 3 (0.14)<br>Tremor: 3 (0.14)<br>Constipation: 2 (0.1)<br>Cough: 2 (0.1)<br>Apnea: 2 (0.1)<br>Atelectasis: 2 (0.1)<br>Cardiac arrest: 2 (0.1)<br>Eye movement disorder: 2 (0.1)<br>Gaze palsy: 2 (0.1)<br>Hypopnea: 2 (0.1)<br>Pneumonitis: 2 (0.1)<br>Respiratory failure: 2 (0.1)<br>Restlessness: 2 (0.1)<br>Seizure: 2 (0.1)<br>Tardive dyskinesia: 2 (0.1)<br>Tongue paralysis: 2 (0.1)<br>Syncope: 2 (0.1)<br>Abdominal pain upper: 1 (0.1)<br>Abdominal pain: 1 (0.1)<br>Aggression: 1 (0.1)<br>Balance disorder: 1 (0.1)<br>Brain edema: 1 (0.1)<br>Breath holding: 1 (0.1)<br>Bronchospasm: 1 (0.1)<br>Death: 1 (0.1)

Diarrhea: 1 (0.1)
Drooling: 1 (0.1)
Dry mouth: 1 (0.1)
Dyskinesia: 1 (0.1)
Eye edema: 1 (0.1)
Fatigue: 1 (0.1)
Feeling cold: 1 (0.1)
Hypoventilation: 1 (0.1)
Mydriasis: 1 (0.1)
Oral pruritus: 1 (0.1)
Psychomotor hyperactivity: 1 (0.1)
Pulmonary edema: 1 (0.1)
Rhonchi: 1 (0.1)
Salivary hypersecretion: 1 (0.1)
Sluggishness: 1 (0.1)
Sneezing: 1 (0.1)
Staring: 1 (0.1)
Tachypnea: 1 (0.1)

Buprenorphine/naloxone film
Lethargy: 23 (20.0)
Respiratory depression: 14 (12.4)
Miosis: 13 (11.5)
Vomiting: 4 (3.5)
Ataxia: 3 (2.6)
Bradycardia: 3 (2.6)
Agitation: 2 (1.7)
Flushing: 2 (1.7)
Bronchospasm: 1 (0.9)
Confusional state: 1 (0.9)
Dysarthria: 1 (0.9)
Gait disturbance: 1 (0.9)
Hypotension: 3 (2.6)
Pallor: 1 (0.9)
Psychomotor hyperactivity: 1 (0.9)

Jabbehdari et al. 2013 [45]	Iran	2012	Female: 15 (48.0)<br>Male: 16 (52.0)	< 12	Methadone: 31 (100.0)	Drowsiness: 23 (75.0)<br>Miotic pupil: 21 (68.0)<br>Vomiting: 19 (61.0)<br>Rapid shallow breathing: 18 (57.0)<br>Apnea: 12 (40.0)	Ingestion
Bazmamoun et al. 2014 [46]	Iran	2007-2013	Female: 28 (45.2)<br>Male: 34 (54.8)	4.5 ± 2.5	Methadone: 62 (100.0)	Decreased consciousness: 57 (91.9)<br>Miotic pupil: 52 (82.3)<br>respiratory depression: 43 (69.4)<br>Seizure: 15 (24.4)<br>Hypotension: 4 (6.5)<br>Aspiration pneumonia: 2 (3.2)<br>Cerebellar edema and hydrocephalus: 1 (1.6)<br>Cerebellar ischemia and secondarily obstructive hydrocephalus: 1 (1.6)<br>Generalized cerebral edema: 1 (1.6)	Ingestion (tablet and syrup)
Sharif and Nouri 2015 [47]	Iran	2009-2013	Female: 25 (43.1)<br>Male: 33 (56.9)	5.2 ± 1.0	Methadone: 58 (100.0)	Drowsiness: 53 (91.4)<br>Miosis: 44 (75.9)<br>Vomiting: 40 (69.0)<br>Ineffective breath (any kind of breathing problem except apnea): 36 (62.1)<br>Apnea: 31 (53.4)<br>Cyanosis: 25 (43.1)<br>Seizure: 5 (8.6)<br>Ataxia: 4 (6.9)<br>Delirium: 2 (3.4)	Syrup form of methadone
Borys et al. 2015 [48]	USA	2008-2013	Female: 52 (50.0)<br>Male: 52 (50.0)	0 - 17	Tapentadol: 104 (100.0)	Drowsiness and lethargy: 30 (29.0)<br>Nausea: 4 (4.0)<br>Vomiting: 4 (4.0)<br>Miosis: 3 (3.0)<br>Tachycardia: 2 (2.0)<br>Respiratory depression: 1 (1.0)<br>Dizziness/vertigo: 1 (1.0)<br>Coma: 1 (1.0)	Not informed

						Dyspnea : 1 (1.0)<br>Edema: 1 (1.0)<br>Hallucinations/delusions: 1 (1.0)<br>Hives/welts: 1 (1.0)<br>Pallor : 1 (1.0)<br>Pruritus: 1 (1.0)<br>Slurred speech: 1 (1.0)	
Hamedi et al. 2016 [49]	Iran	2015	Female: 34 (43.0)<br>Male: 45 (57.0)	Female: 5.0 ± 3.4<br>Male: 4.4 ± 3.2	Methadone: 79 (100.0)	Drowsiness: 73 (92.4)<br>Bradypnea: 66 (83.5)<br>O_2 saturation level: 52 (75)<br>Nausea and vomiting: 50 (63.3)<br>Miotic pupil 51 (64.6)<br>Itching: 34 (43.0)	Accidentally by children themselves: 42 (53.0)<br>Accidentally given to children by parents: 28 (35.0)<br>Used for treatment: 4 (5.0)<br>Unknown source: 4 (5.0)<br>Suicide: 1 (1.0)
Stassinos et al. 2017 [50]	USA	2000-2013	Female: 3447 (47.0)<br>Male: 3887 (53.0)	~ 2	Tramadol: 7334 (100.0)	Drowsiness/lethargy: 611 (8.3)<br>Vomiting: 178 (2.43)<br>Ataxia: 59 (0.80)<br>Tachycardia: 58 (0.79)<br>Miosis: 57 (0.78)<br>Agitated/irritable: 51 (0.70)<br>Respiratory depression/arrest: 36 (0.5)<br>Seizures: 24 (0.3)<br>Hypertension: 11 (0.1)<br>Bradycardia: 8 (0.1)<br>Coma: 5 (0.1)<br>Cyanosis: 4 (0.1)<br>Hypotension: 3 (0.1)<br>Dizziness: 21 (0.2)<br>Pruritus: 19 (0.2)<br>Confusion: 11 (0.1)<br>Hallucinations/delusions: 11 (0.1)	Unintentional: 7186 (98.0)<br>Therapeutic errors: 105 (1.4)<br>Other/unknown reason: 43 (0.6)

						Tremor: 10 (0.1)<br>Mydriasis: 9 (0,1)<br>Diaphoresis: 5 (0.1)<br>Dystonia: 5 (0.1)	
Toce et al. 2017 [51]	USA	2006-2014	Female: 43 (49.0)<br>Male: 45 (51.0)	~ 2	Buprenorphine/naloxone: 86 (98.0)<br>Buprenorphine: 2 (2.0)	Respiratory depression: 83 (94.3)<br>Depressed mental status (lethargic, somnolent, sleepy, drowsy): 80 (90.9)<br>Miosis: 77 (87.5)<br>SpO_2 <93% : 28 (31.8)<br>Agitation: 5 (5.7)<br>Emesis: 4 (4.5)	Not informed
Carreiro et al. 2019 [52]	USA	2010-1016	Female + Male: 773	< 18	Buprenorphine: 135 (17.5)<br>Oxycodone: 131 (17.0)<br>Hydrocodone: 108 (14.0)<br>Tramadol: 107 (13.8)<br>Methadone: 83 (10.7)<br>Heroin: 66 (8.5)<br>Morphine: 47 (6.0)<br>Codeine: 40 (5.2)<br>Other/Not Specified: 37 (4.8)<br>Fentanyl: 19 (2.5)	Adverse cardiovascular events (ACVE) (myocardial injury, shock, ventricular dysrhythmia, or cardiac arrest)<br>Buprenorphine: 1<br>Oxycodone: 8<br>Hydrocodone: 2<br>Tramadol: 5<br>Methadone: 7<br>Heroin: 6<br>Morphine: 5<br>Codeine: 2<br>Other/Not Specified: 3<br>Fentanyl: 1	Not informed
Riasi et al. 2019 [53]	Iran	2015-2017	Female: 40 (48.2)<br>Male: 44 (51.8)	3.46 ± 3.36	Methadone: 33 (38.8)<br>Opium or other opioids: 52 (61.2)	Altered consciousness: 76 (89.4)<br>Confused: 46 (54.1)<br>Respiratory acidosis: 45 (52.9)<br>Vomiting: 40 (47.1)<br>Dyspnea: 34 (40.0)<br>Lethargic: 25 (29.4)<br>Cyanosis: 22 (25.9)	Methadone syrup: 21 (24.7)
Farnaghi et al. 2021 [54]	Iran	2018-2019	Methadone<br>Female: 35 (38.9)<br>Male: 55 (61.1)	< 12	Methadone: 90 (75.0)<br>Buprenorphine: 30 (25.0)	Bradypnea<br>Methadone: 39 (43.3)<br>Buprenorphine: 10 (33.3)	Ingestion

			Buprenorphine<br>Female: 16 (53.0)<br>Male: 14 (47.0)			Apnea<br>Methadone: 20 (22.2)<br><br>Miosis<br>Methadone: 79 (88.0)<br>Buprenorphine: 17 (57)<br><br>Vomiting<br>Methadone: 48 (53.3)<br>Buprenorphine: 17 (56.6)<br><br>Itching<br>Methadone: 26 (28.8)<br>Buprenorphine: 15 (50.0)	
Cohen et al. 2022 [55]	USA	2017-2020	Females: 87 (52.7)<br>Male 78 (47.3)	9.1 ± 6.8	Buprenorphine: 31 (19.0)<br>Fentanyl: 28 (16.9)<br>Oxycodone: 31(19.0)<br>Methadone: 12 (7.0)<br>Unknown: 66 (40.0)	Central Nervous System depression: 115 (70.0)<br>Opioid toxidrome: 66 (40.0)<br>Respiratory depression: 65 (39.0)<br>Bradypnea: 27 (16.0)<br>Hypotension: 12 (7.0)	Ingestion: 133 (81.0)<br>Intravenous: 2 (1.0)<br>Intranasal/inhalation: 5 (3.0)<br>Sublingual: 4 (2.0)<br>Other/unknown: 21 (13.0)
Caré et al. 2022 [56]	France	2003-2019	Female: 40 (27.6)<br>Male: 105 (72.4)	3.0 [1.9; 4.0]	Tramadol 145 (100.0)	Drowsiness: 52 (35.9)<br>Vomiting: 21 (14.5)<br>Myosis: 16 (11.0)<br>Significant bradypnea: 12 (8.3)<br>Ataxia: 7 (4.8)<br>Acute urine retention: 6 (4.1)<br>Moderate decrease in consciousness: 6 (4.1)<br>Abdominal pain: 5 (3.4)<br>Agitation: 5 (3.4)<br>Brief apnea: 4 (2.8)<br>Confusion: 4 (2.8)<br>Nausea: 4 (2.8)<br>Diaphoresis: 3 (2.1)	Ingestion

	Prolonged vomiting: 4 (2.8)<br>Mydriasis: 3 (2.1)<br>Sinus tachycardia: 3 (2.1)<br>Tremor or hypertonia: 3 (2.1)<br>Coma: 2 (1.4)<br>Skin rash/pruritus: 2 (1.4)<br>Vertigo: 2 (1.4)<br>Blurred vision: 1 (0.7)

Table S5 of supplementary material. Summary of systematically selected studies from 2011 to 2024 that evaluated the harmful effects of opioids in the general population (Group 2).

Reference	Country	Follow up	Sample characteristics		Harmful effects		
			Sex [N (%)]	Age	Drugs [N (%)]	Clinical symptoms [N (%)]	Type of exposure
Emamhadi et al. 2012 [57]	Iran	2009-2011	Female: 107 (22.3)<br>Male: 372 (77.7)	22.6 ± 6 years	Tramadol: 479 (100.0)	Seizures	Ingestion
Eizadi-Mood et al. 2014 [58]	Iran	2010-2011	Female: 34 (32.7)<br>Males: 70 (67.3)	26.3 ± 9 years	Tramadol: 104 (100.0)	Confusion: 31 (29.8)<br>Loss of consciousness: 26 (25)<br>Vomiting: 17 (16.3)<br>Nausea: 15 (14.4)<br>Need to intubation: 14 (14.0)<br>Seizure: 13 (12.5)<br>Aspiration pneumonia: 4 (4.0)<br>Hypotension: 4 (4.0)<br>Tachycardia: 4 (4.0)<br>Hyperventilation: 3 (3.0)<br>Apnea: 3 (3.0)<br>Renal failure: 3 (3.0)<br>Coma: 1 (1.0)	Not informed
Asadi et al. 2015 [59]	Iran	2012	Female: 15 (17.9)<br>Male: 69 (82.1)	26.4 ± 6.5 years	Tramadol: 84 (100.0)	Seizure	Not informed
Farzaneh et al.2016 [60]	Iran	2014-2015	Female: 35 (19.4)<br>Male: 145 (80.6)	< 20 years old: 27 (15.0)<br>20-30 years old: 68 (37.8)<br>30-40 years old: 41 (22.8)<br>40-50 years old: 23 (12.7)<br>> 50 years old: 21 (11.7)	Tramadol: 75 (41.7)<br>Methadone: 31 (17.2)<br>Illicit: 74 (41.1)	Asthma<br>Hypertension<br>Muse<br>Nausea and vomiting<br>Restlessness<br>Urinary Retention<br><br>*The article doesn't specify the symptoms per opioid type and doesn't specify if illicit or licit	Not informed

						opioids caused the greatest damages	
Ghamsari et al.2016 [61]	Iran	2012 - 2013	Female: 405 (28.9)<br>Male: 997 (71.1)	24 ± 6 years	Tramadol: 1402 (100)	Sinus tachycardia: 463 (33)<br>Dominant S wave in either I or aVL lead: 395 (28.1)<br>Right axis deviation: 340 (24.2)<br>Long QTc interval: 259 (18.4)<br>QRS widening: 91 (6.5)<br>Right bundle branch block: 73 (5.2)<br>Sinus bradycardia: 1 (0.1)	Not informed
Moghadam et al.2016 [62]	Iran	2013-2014	Low dose group<br>Female: 2 (2.7)<br>Male: 68 (97.3)<br><br>High dose group<br>Female: 13 (14.6)<br>Male: 76 (85.4)	Low dose group 25.8 ± 7.3 years<br><br>High dose group 22.0 ± 6.2 years	Tramadol 159 (100.0)	Seizure: 159 (100.0)<br>Sinus tachycardia: 68 (42.5)<br>Sinus bradycardia: 4 (2.4)	Not informed
Ahmadimanesh et al. 2018 [63]	Iran	2017	Female: 29 (24.0)<br>Male: 91 (76.0)	22.8 ± 5.8 years	Tramadol 120 (100.0)	Seizure: 50 (42.0)	Not informed
Bedson et al. 2018 [64]	United Kingdon	2002-2012	Female: 57,937 (59.0)<br>Male: 40,203 (41.0)	61 (47.7) years	Morphine and no discriminated opioids 98,140	Addiction falls<br>Accidental harmful effects<br>Gastrointestinal pathology<br>Gastrointestinal bleeding<br>Anemia	Not informed
Mohammadpouret al. 2019 [65]	Iran	2016-2017	Female: 39 (32.3)<br>Male: 82 (67.7)	~ 25 years	Tramadol: 121 (100.0)	Seizures<br>Tremors<br>Severe muscle spasms<br>Kidney and heart damage<br><br>*Following harmful effects and seizures, shivering, severe muscle contractions, and tissue damage occurred, especially in the heart and kidney.	Not informed

Ahmadimanesh et al. 2020 [66]	Iran	2017	Female: 23 (24.0)<br>Male: 73 (76.0)	Female:<br>21.9 ± 5.5<br>Male:<br>22.8 ± 5.7	Tramadol: 96 (100.0)	Coma<br>Headache<br>Hypertension<br>Nausea<br>Respiratory depression<br>Seizure<br>Tachycardia<br>Vertigo<br>Vomiting	Ingestion
Caré et al. 2023 [67]	French	2011-2020	*French National Database of Poisonings*<br>Female 297 (76.5)<br>Male 91 (23.5) French *Pharmacovigilance Network Database Female* 113 (72.9) Male 42 (27.1)	*French National Database of Poisonings*<br>40 (29–52.8)<br><br>*French Pharmacovigilance Network Database*<br>41 (30–55)	*French National Database of Poisonings*<br>Tramadol 287 (74)<br>Codeine 101 (26)<br><br>French Pharmacovigilance Network Database<br>Tramadol 87 (56.1)<br>Codeine 60 (38.7)	*French National Database of Poisonings*<br>**Codeine:**<br>Nausea and vomiting: 49 (48.5)<br>Abdominal pain: 37 (36.6)<br>Asthenia; 11 (10.9)<br>Dizziness: 48 (47.5)<br>Drowsiness: 14 (13.9)<br>Skin eruption: 6 (5.9)<br>Tremor: 6 (5.9)<br>Presyncope or syncope: 6 (5.9)<br>Dyspnea: 4 (4.0)<br>Pruritus: 4 (4.0)<br>Hyperhidrosis: 3 (3.0)<br>Paresthesia: 3 (3.0)<br>Cognitive impairment: 2 (2.0)<br>Headache: 2 (2.0)<br>Palpitations: 2 (2.0)<br>Angioedema and anaphylaxis: 1 (1.0)<br>Constipation: 1 (1.0)<br>Feeling of chest constriction: 1 (1.0)<br>Flushing: 1 (1.0)<br><br>**Tramadol**	Ingestion

	Nausea and vomiting: 231 (80.5)<br>Dizziness: 211 (73.5)<br>Presyncope or syncope: 30 (14.5)<br>Asthenia: 44 (15.3)<br>Drowsiness: 43 (15.0)<br>Headache: 40 (13.9)<br>Tremor: 30 (10.5)<br>Hyperhidrosis: 23 (8.0)<br>Pruritus: 17 (5.9)<br>Palpitations: 12 (4.2)<br>Abdominal pain: 9 (3.1)<br>Flushing: 9 (3.1)<br>Paresthesia: 8 (2.8)<br>Dyspnea: 7 (2.4)<br>Tinnitus: 5 (1.7)<br>Dry mouth: 4 (1.4)<br>Blurred vision: 3 (1.0)<br>Cognitive impairment; 3 (1.0)<br>Hallucination: 3 (1.0)<br>Seizure: 2 (0.7)<br>Skin eruption: 2 (0.7)<br>Diplopia: 1 (0.3)<br>Euphoric mood: 1 (0.3)<br>Taste altered: 1 (0.3)<br><br>*French Pharmacovigilance Network Database*<br>**Codeine:**<br>Abdominal pain 24 (40.0)<br>Nausea and vomiting 15 (25.0)<br>Dizziness 11 (18.3)<br>Acute pancreatitis 9 (15.0)

Angioedema and anaphylaxis 8 (13.3)
Dyspnea 7 (11.7)
Presyncope or syncope 7 (11.7)
Respiratory 7 (11.7)
Urticaria 6 (10.0)
Pruritus 5 (8.3)
Skin eruption 5 (8.3)
Stiffness 3 (5.0)
Tremor 3 (5.0)
Asthenia 2 (3.3)
Blurred vision 2 (3.3)
Drowsiness 2 (3.3)
Palpitations 2 (3.3)
Paresthesia 2 (3.3)
Appetite decreased 1 (1.7)
Chills 1 (1.7)
Concentration impaired 1 (1.7)
Confusion 1 (1.7)
Euphoric mood 1 (1.7)
Flushing 1 (1.7)
Hallucination 1 (1.7)
Headache 1 (1.7)
Hyperhidrosis 2 (3.3)
Hypertension 1 (1.7)
Hypotension 1 (1.7)
Insomnia 1 (1.7)
Dry mouth: 5 (5.7)
Hiccups: 1 (1.1)
Nausea and vomiting: 45 (51.7)
Urinary retention: 1 (1.1)
Stiffness: 3 (3.4)
Tremor: 8 (9.2)
Blurred vision: 5 (5.7)

Nystagmus: 1 (1.1)
Dyspnea: 9 (10.3)

Tramadol
Nausea and vomiting: 45 (51.7)
Dizziness: 30 (34.5)
Presyncope or syncope: 20 (23.0)
Asthenia: 14 (16.1)
Hyperhidrosis: 14 (16.1)
Abdominal pain: 13 (14.9)
Headache: 11 (12.6)
Dyspnea: 9 (10.3)
Pruritus: 8 (9.2)
Tremor: 8 (9.2)
Flushing: 7 (8.0)
Drowsiness: 7 (8.0)
Palpitations: 6 (6.9)
Blurred vision: 5 (5.7)
Dry mouth: 5 (5.7)
Cognitive impairment: 5 (5.7)
Constipation: 4 (4.6)
Hypotension: 4 (4.6)
Insomnia: 4 (4.6)
Concentration impaired: 3 (3.4)
Confusion: 3 (3.4)
Diarrhea: 3 (3.4)
Paresthesia: 3 (3.4)
Stiffness: 3 (3.4)
Angioedema and anaphylaxis: 2 (2.3)
Appetite decreased: 2 (2.3)

Euphoric mood: 2 (2.3)<br>Skin eruption: 2 (2.3)<br>Feeling of chest constriction: 1 (1.1)<br>Hallucination: 1 (1.1)<br>Hiccups: 1 (1.1)<br>Nystagmus: 1 (1.1)<br>Tinnitus: 1 (1.1)<br>Abnormal dreams: 1 (1.1)<br>Acute pancreatitis: 1 (1.1)<br>Urinary retention: 1 (1.1)<br>Depersonalization: 1 (1.1)

	Bias due to confounding	Bias in selection of participants into the study	Bias in measurement of interventions	Bias due to departure from intended intervention	Bias due to missing data	Bias in measurement of outcomes	Bias in selection of reported results	Overall risk of bias
Pedapati and Bateman 2011	+	+	+	+	+	+	+	Low
Lavonas et al 2013	+	-	+	+	?	+	+	Serious
Jabbehdari et al 2013	+	+	+	+	+	?	+	Moderate
Bazmamoun et al. 2014	+	+	+	+	+	?	?	Moderate
Sharif and Nouri 2015	+	+	+	+	+	+	+	Low
Borys et al 201	+	-	+	+	?	?	?	Serious
Hamedi et al. 2016	+	?	+	?	?	+	+	Moderate
Stassinos et al. 2017	+	?	+	+	?	?	?	Moderate
Toce et al. 2017	+	?	+	+	?	?	?	Moderate
Carreiro et al. 2019	+	-	+	+	?	+	+	Serious
Riasi et al. 2019	+	?	+	+	?	+	+	Moderate
Farnaghi et al 2021	+	?	+	+	?	+	+	Moderate
Emamhadi et al 2012	+	-	?	+	?	+	+	Serious
Eizadi-Mood, et al 2014	+	?	+	+	+	?	+	Moderate
Asadi et al 2015	+	?	+	+	?	+	+	Moderate
Farzaneh et al 2016	+	?	+	+	?	+	+	Moderate
Ghamsari et al 2016	+	+	+	+	+	+	+	Low
Moghadam et al 2016	+	+	+	+	+	+	+	Low
Ahmadimanesh et al 2018	+	+	+	+	+	?	?	Moderate
Bedson et al 2018	+	?	+	+	?	+	+	Moderate
Mohammadpouret al 2019	+	+	+	+	+	?	?	Moderate
Ahmadimanesh et al 2020	+	+	+	+	+	+	+	Low
Cohen et al 2022	+	+	?	+	?	?	+	Moderate
Caré et al 2022	+	+	+	+	-	+	+	Moderate
Caré et al 2023	+	+	?	+	?	?	+	Moderate

Figure S1 Risk of bias of the 25 selected articles.
